# Resonantly Enhanced Betatron Hard X-rays from Ionization Injected Electrons in a Laser Plasma Accelerator

**DOI:** 10.1038/srep27633

**Published:** 2016-06-08

**Authors:** K. Huang, Y. F. Li, D. Z. Li, L. M. Chen, M. Z. Tao, Y. Ma, J. R. Zhao, M. H. Li, M. Chen, M. Mirzaie, N. Hafz, T. Sokollik, Z. M. Sheng, J. Zhang

**Affiliations:** 1 Beijing National Laboratory of Condensed Matter Physics, Institute of Physics, CAS, Beijing 100190, China; 2 Institute of High Energy Physics, CAS, Beijing 100049, China; 3 Collaborative Innovation Center of IFSA, Shanghai Jiao Tong University, Shanghai 200240, China; 4 Key Laboratory for Laser Plasmas (MOE) and Department of Physics and Astronomy, Shanghai Jiao Tong University, Shanghai 200240, China; 5 SUPA, Department of Physics, University of Strathclyde, Glasgow G4 0NG, United Kingdom

## Abstract

Ultrafast betatron x-ray emission from electron oscillations in laser wakefield acceleration (LWFA) has been widely investigated as a promising source. Betatron x-rays are usually produced via self-injected electron beams, which are not controllable and are not optimized for x-ray yields. Here, we present a new method for bright hard x-ray emission via ionization injection from the K-shell electrons of nitrogen into the accelerating bucket. A total photon yield of 8 × 10^8^/shot and 10^8 ^photons with energy greater than 110 keV is obtained. The yield is 10 times higher than that achieved with self-injection mode in helium under similar laser parameters. The simulation suggests that ionization-injected electrons are quickly accelerated to the driving laser region and are subsequently driven into betatron resonance. The present scheme enables the single-stage betatron radiation from LWFA to be extended to bright γ-ray radiation, which is beyond the capability of 3^rd^ generation synchrotrons.

Ultrafast x-ray sources have tremendous applications in time-resolved x-ray diffraction and x-ray absorption spectroscopy to study the transient properties of condensed matter and biological structures^1^,^2^. These sources have mainly been achieved by x-ray free electron lasers (XFELs) with high brightness^3^,^4^. However, XFELs are huge facilities that are only accessible to limited users. With the development of femtosecond, high-power lasers^5^, laser plasma x-ray sources are becoming increasingly attractive owing to their compactness and the natural synchronization of the drive lasers and the produced x-ray sources. X-ray emission from laser plasma interactions, such as Kα x-ray emission, nonlinear Thomson scattering and betatron x-ray sources, have been intensively studied^6^,^7^,^8^,^9^,^10^,^11^,^12^,^13^,^14^. In particular, the betatron x-rays generated from electron oscillations in laser wakefield acceleration (LWFA) are a promising source owing to the high spatial coherence^12^, high photon yield (>10^8^/shot) and high photon energy (up to MeV)^13^.

Electrons trapped inside the laser wakefield are accelerated longitudinally^15^ and undergo betatron oscillation owing to the presence of transverse electric and magnetic fields in the wakefield^14^,^16^,^17^. The oscillation period of the betatron motion in LWFA is thousands of times smaller than that of the conventional magnetic wiggler. Therefore, betatron x-rays generated from LWFA can reach the hard x-ray or γ-ray region^18^. Betatron radiation is emitted in regimes distinguished by the wiggling strength parameter, defined as 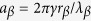, where 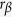 and *λ*_*β*_ are the betatron oscillation amplitude and wavelength, respectively^19^. In the case of LWFA, where α_*β*_≫1,the emitted x-ray beam has a synchrotron like broadband spectrum, which can be expressed as 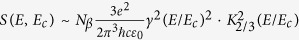, where 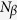 is the number of oscillations, *K*_*2/3*_ is a modified Bessel function of the second kind and 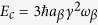 is the critical energy(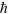
is the reduced Planck constant and *ω*_*β*_is the betatron frequency). The spectrum peaks at *E*~0.5*E*_c_, beyond which the radiation tends to decay exponentially^12^.

The study of electron acceleration and the resultant betatron x-ray emission from LWFA usually utilizes low Z gases in the self-injection regime. The betatron photon properties have varied with different laser parameters and plasma densities in previous experiments^11^,^12^,^13^,^14^. In the case of non-resonance, electron beams are accelerated in the wakefield with high stability, and the betatron source size can reach a few μm^12^,^20^, with low photon yield and photon energy. By contrast, if the acceleration procedure is extended to the case of resonance, the electron beam will catch up with the laser and oscillate with the laser field, which results in the photon energy entering the γ-ray region^13^. However, in the mode of self-injection, the electron beam will experience a long acceleration period before it catches the laser pulse, resulting in the uncontrollability of the x-ray generation. Real applications require the production of x-ray emission with a sufficient number of photons and suitable radiation spectra. Therefore, the control of the accelerated electron energy, total charge and betatron oscillation dynamics is highly important. Ionization injection in the LWFA has the advantage of reduced laser intensity thresholds and better injection control compared with self-injection, even though the energy spread of the produced beams must be continuously improved^21^,^22^,^23^,^24^,^25^,^26^,^27^. Resonantly excited betatron x-ray emission via ionization-injected electron beams has not yet been investigated in experiments.

In this work, we report the first study of bright, hard x-rays based on ionization-injected electron beams accelerated in LWFA via betatron oscillations. Highly collimated hard x-rays with a photon flux of 8 × 10^8^/shot and with 10^8^ photons greater than 110 keV have been produced with a pure nitrogen gas jet irradiated with 100 TW laser pulses. This yield is approximately 10 times higher than that obtained with helium gas under similar laser conditions, and much higher than other experimental results reported for the self-injection mode. Two-dimensional (2D) particle-in-cell (PIC) simulations suggest that the enhanced betatron photon energy and photon flux are due to ionized early injection and effective betatron resonant oscillation of electrons in the laser fields.

## Results

### Experimental setup

**Figure 1:**
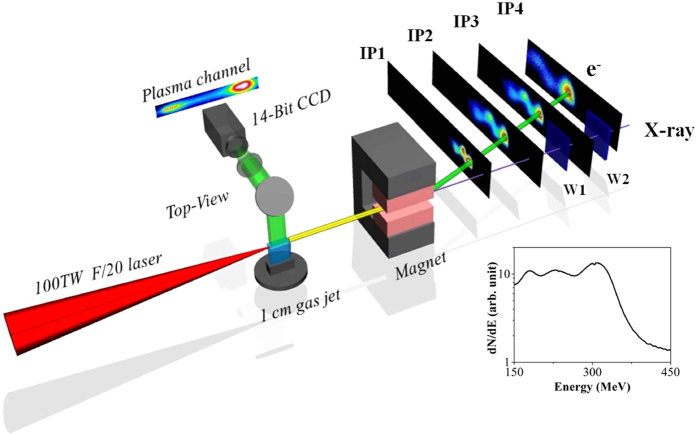
Experimental setup. All IPs are wrapped with 13 μm Al foil. Two tungsten filters with thicknesses of W1 = 25 μm and W2 = 56 μm are placed, respectively, between IP2 and IP3 and between IP3 and IP4 on the laser axis to attenuate the x-ray beam. The consecutive IPs show the dispersed electron bunch. A sample of the plasma channel is shown in the inset above the CCD. The inset in the lower right corner is an example of the experimental electron energy spectrum for nitrogen.

The experimental setup is shown in Fig. 1. The laser pulses from a 100 TW laser system were focused by an off-axis-parabola mirror onto a 1.2 mm × 10 mm supersonic gas jet (see Methods for details). For comparison, we separately used N_2_ and He gases in the experiment. A top-view system, including a charge-coupled device (CCD) and a low-pass filter, was used to monitor Thomson-scattering, which can reveal the interaction position and the time-integrated plasma channel length.

### Experimental results

**Figure 2:**
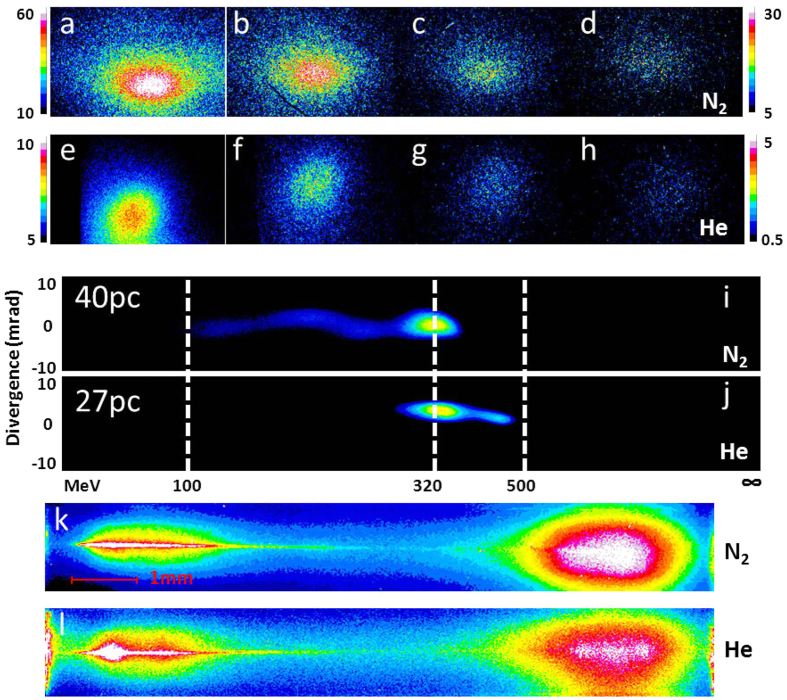
Experimental results. (**a–d**,**e–h**) are the betatron x-ray signals with nitrogen and helium gases, respectively, on IPs 1–4; (**i,j**) show the electron energy spectra with nitrogen and helium gases, which are shown in the same color scale (the charges denote electrons with energy greater than 100 MeV); (**k,l**) show the plasma channel images with nitrogen and helium gases, respectively.

Fig. 2 shows the experimental betatron x-ray profile (a–d, e–h) corresponding to the electron spectra signal (i, j) and the laser plasma channel length (k, l) for different gas species. By integration of the x-ray signal, we find that the betatron x-ray emission from nitrogen plasma (a–d) is 10-fold the emission from the helium plasma (e–h) for x-rays with energy >7 keV. The profiles of the betatron signals from the nitrogen gas exhibit a clear elliptical structure with divergence angles of 6 mrad and 3 mrad in FWHM along the long axis and short axis, respectively. The long axis is along the laser polarization, which indicates that the betatron oscillations of the electrons are asymmetric in the transverse directions. The background electron plasma densities for nitrogen and helium are 3.0 × 10^18 ^cm^−3^ and 4.6 × 10^18 ^cm^−3^, respectively, at which the betatron signals are the strongest from each gas. At a plasma density of 3.0 × 10^18 ^cm^−3^ for helium, no electron signal was observed, which suggests that the electron beams from nitrogen gas are associated with the K-shell ionization- injection process. The electron signal with the nitrogen gas in Fig. 2(i) has an almost continuous spectrum with a spectral peak at approximately 320 MeV and a low energy tail. The electron spectrum of the helium plasma was also almost continuous with a similar energy peak, but the maximum energy reached 500 MeV. As shown in Fig. 2(k,l), the laser plasma channel lengths with both gas species are approximately the same (~9 mm). Therefore, with similar channel length and lower maximum energy, the betatron x-ray yield and photon energy generated from nitrogen were much higher than from helium. This comparison between the two gases is important to understand the mechanism responsible for the enhanced betatron x-ray yield and photon energy from the nitrogen gas, as discussed later.

**Figure 3:**
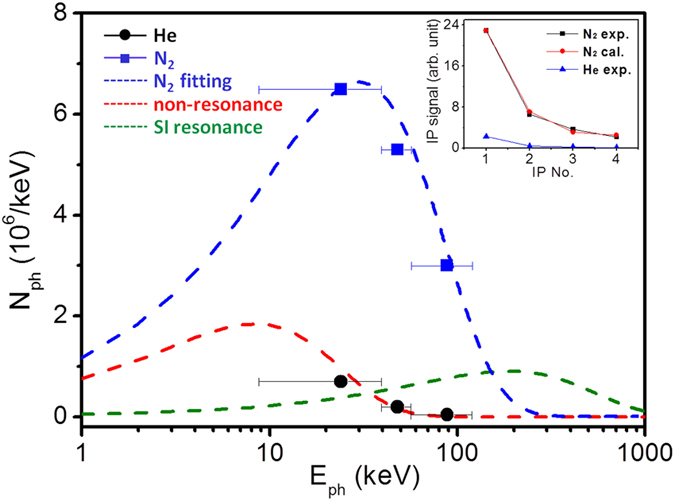
X-ray spectrum analysis. The blue squares and black circles denote the measured average photon number produced with nitrogen and helium gases, respectively. The blue dashed line shows the fitted spectrum for nitrogen gas with the critical energy of 75 keV. The red and green dashed lines represent the x-ray spectra from refs 12 and 13, which correspond to self-injection (SI) non-resonance and resonance. The inset in the figure shows the x-ray signal strength on consecutive IPs. The black squares and red circles represent the experimental signal and the calculated value using the synchrotron radiation distribution, respectively. The blue triangle shows the experimental x-ray signal with helium gas.

The spectra of the generated betatron x-rays are shown in Fig. 3. The average photon flux per keV can be estimated for different photon energy ranges, which are separated by the cut-off energy on different image plates (IPs). The average photon numbers for nitrogen or helium gas are plotted explicitly. For comparison, the detected x-ray spectra from previous work^12^,^13^ are also shown. The x-rays generated from helium in our experiment have a similar spectrum to that of ref. 12 at similar laser intensity. However, the x-ray source we obtained from the nitrogen gas has a much higher photon yield and reaches a much higher photon energy. With the much lower laser power and electron energy in the present case, the photon energy is not as high as that generated in ref. 13. The nitrogen x-ray source in our case has a much higher photon yield that peaked at 30–40 keV, which is in the hard x-ray region. The relative signal strength of the betatron x-ray on IPs 1–4 is illustrated in the inset of Fig. 3, representing the relative experimental x-ray signal strength with nitrogen and helium gases, respectively. The nitrogen betatron x-ray signal is fitted using a least squares fitting method assuming a synchrotron-like spectrum^28^. The best fit is found with the critical energy of E_c_ = 75 keV. With a similar electron energy, the nitrogen betatron x-ray critical energy is more than three times that in previous work with Helium gas^12^,^14^. Our spectrum shows an energy peak at 37 keV, where the photon flux is approximately 6.5 × 10^6^ photons/keV. The total x-ray yield on the spectrum reaches 8 × 10^8^/shot. By integration of the x-ray signal on the last IP, the photon number between 110 keV~1 MeV is estimated to be 10^8^, which is in good agreement with the fitted spectrum.

### Simulations results

To understand the mechanisms of the enhanced betatron radiation in nitrogen gas, 2D-PIC simulations were conducted (see Methods). For comparison, simulations were also performed for helium gas. A set of laser and plasma parameters were tested to investigate the electron injection and acceleration mechanism for separate gases. The experiments and simulations showed that with the same laser parameter and background plasma density as those used for nitrogen in optimized x-rays, there were no electron beams generated from the helium gas. To compare the electron behavior of helium and nitrogen, we used a higher plasma density to achieve the self-injection condition for helium gas, which is in correspondence with the conditions of the experimental results. The transverse evolution of the laser pulse and the electron trapping efficiency should be different in the 3D simulation compared with the 2D simulation. Additionally, the x-ray angular distribution and spectra could not be quantitatively deduced from the 2D simulations. Therefore, on the basis of the experimental results, the simulation presented here is used to qualitatively elaborate the advantage of using nitrogen gas to enhance betatron x-ray generation.

**Figure 4:**
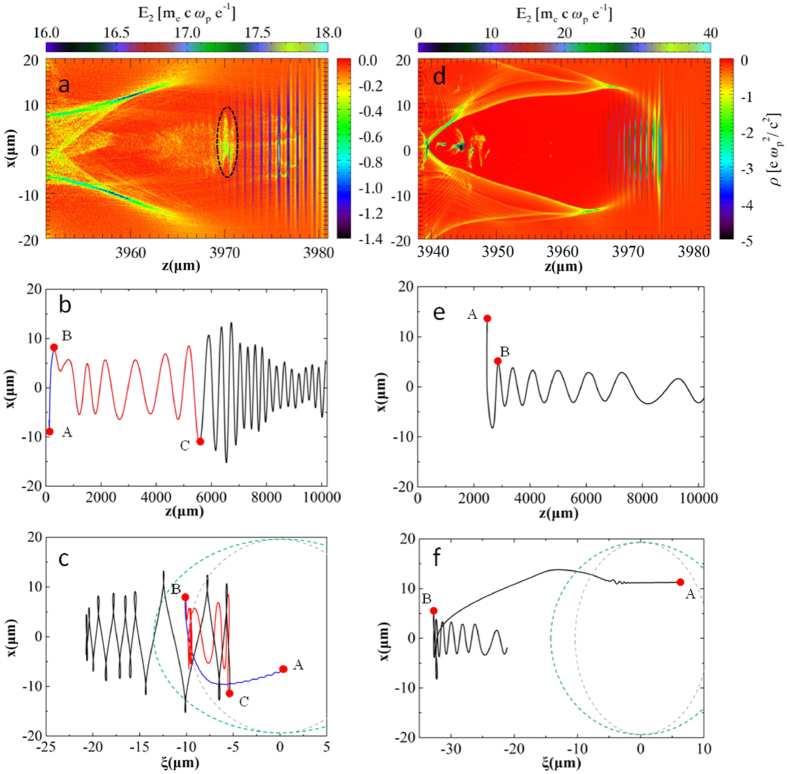
PIC simulation results. (**a**) Electron density distribution together with the laser pulse, where the black dashed elliptical circle marks the portion of electrons performing resonant betatron oscillations. (**b**) Trajectory of a test electron within the black dashed circle in the laboratory reference. (**c**) The same test electron in the reference frame of laser (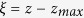, where 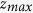 is the location of the peak laser field). A, B and C denote the electron birth position (released from a nitrogen ion), injection position, and resonant oscillation transition point. The injection, weakly resonant, and strong resonant oscillation processes are shown in blue, red, and black lines, respectively. (**d**), (**e**), (**f**) The corresponding plots for helium. The bubble size in Fig. 4(d) is a little larger than in Fig. 4(a), which is due to the stronger laser self-focusing and self-steepening effects for helium.

In Fig. 4(a), large amplitudes of betatron oscillation of the trapped electrons are observed after 4 mm propagation of the laser pulse in the nitrogen gas. The trapped electron beam is spatially distributed in the region of the driving laser and is modulated periodically with a period equal to the local laser wavelength of 0.8 μm. However, the electrons in helium gas are injected at the tail of the wakefield bucket, which is far from the laser pulse, as is shown in Fig. 4(d). The black dashed elliptical circle in Fig. 4(a) highlights this portion of the electrons, which has a bunch length ~10 fs. A typical trajectory of a test electron located within this portion of the trapped bunch in the laboratory frame is shown in Fig. 4(b). For betatron movement in the bubble without the presence of a laser field, the period of electron oscillation 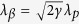 would increase with acceleration. However, this result is not found in Fig. 4(b). The electron firstly experiences small amplitude betatron oscillation due to the original off-axis injection and the restoring force of the wakefield. Then, because the laser pulse partially fills the bubble, the oscillation amplitude is gradually increased during the electron acceleration as a result of weakly interacting with the laser pulse. This process is known as the weak-resonant case. Finally, the electron is accelerated towards the driving laser pulse, and the transverse oscillation amplitude r_0_ is suddenly increased to larger than 10 μm when it is resonant with the laser pulse. Meanwhile, the oscillation period is decreased to 400 μm. However, in the case of helium shown in Fig. 4(e), the electron transverse oscillation amplitude is damped, and the oscillation period is increased as the electron accelerates, which was the typical electron trajectory in the wakefield^29^ when no interference with the laser pulse occurred.

The resonant betatron oscillation condition is written as 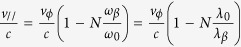. At the electron location of 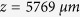, the test electron has an energy of 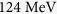 (γ = 243), corresponding to 
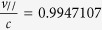
. At the given plasma density profile and laser intensity, the laser has a phase velocity of 
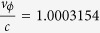
, which is determined by measuring the nonlinear index at the location of the electron in the simulation. The betatron oscillation period is 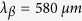 at this point in the simulation. The parameters fit well with a harmonic number of N = 4. We note that in our case, the electron was harmonically resonant with the laser, where N > 1 instead of N = 1. In a realistic laser wakefield acceleration process, the electron energy is always changing. To obtain resonant oscillation, the betatron period should be correspondingly modified. A similar case was observed in ref. 13. The high oscillation frequency, large oscillation amplitude and much greater oscillation period result in the enhancement of the betatron photon energy and photon yield. This result is also proved by the elliptical shape of the radiation distribution observed in the experiments, which suggests an overlap of the laser electromagnetic field and the accelerated electron beam.

## Discussion

To gain insight into the injection and acceleration of ionization injection, the trajectory of the same electron shown in Fig.4(b) is plotted in a reference frame of the light pulse, as shown in Fig. 4(c). The electron born at point A near the peak of the laser field (ξ~0) firstly travels backward with respect to the laser pulse to point B (via blue line) where it is injected. The nonlinear plasma wavelength is 25 μm in the present simulation, and the electron injection point B is 20 μm from the front of the bubble and 10 μm from the laser peak. Therefore, the electron is injected well before it moves to the tail of the bubble and is very close to the laser pulse. Thus, the injected electron can quickly catch the laser pulse (marked by the red line) and experience weak resonant oscillation during the process. At point C, the electron starts to move backward, oscillating with large amplitudes, and the trajectory is the typical resonant oscillation style forced by the laser electromagnetic field^30^ (via the black line). However, in Fig. 4(f), the test electron in helium gas experiences much longer drift along the bubble shell in the wakefield before injection. The electrons are injected at 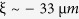, which is far from the laser pulse and is close to the tail of the wakefield bucket. After 1 cm propagation, there is no laser electron coupling, and the electron oscillation amplitude is small. The dashed grey (green) circles in Fig. 4(c,f) are sketches of the laser pulse at different times, corresponding to t = 0 (and t = 15 ps).

**Figure 5:**
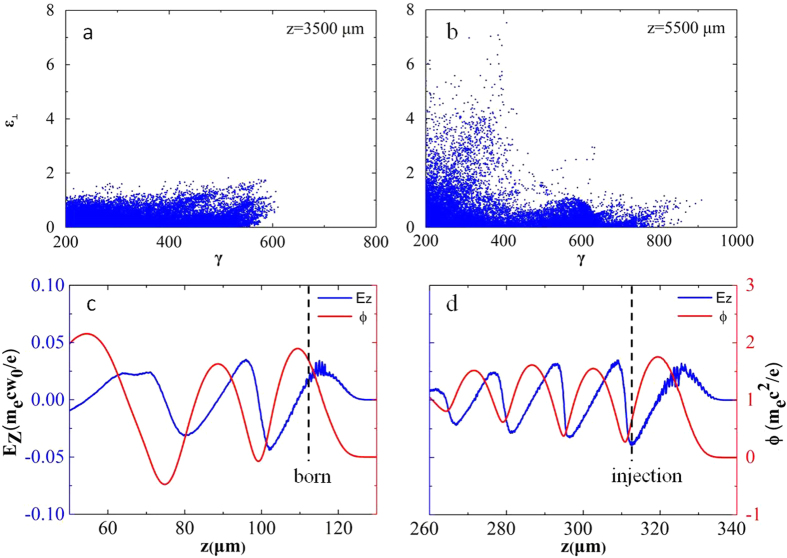
(**a**,**b**) are the 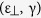 space of the accelerated electrons at different laser propagation distances in nitrogen, corresponding to z = 3500 μm and 5500 μm. (**c** and **d**) show snapshots of the longitudinal wakefield potential (red line) and strength (blue line) at the time when the test electron is born and injected, where the black dashed lines in the two plots mark the electron birth and injection positions, respectively.

To explicitly illustrate the laser electron coupling process, we plot the 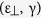 space of the accelerated electrons at different laser propagation distances in nitrogen, where 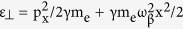 is the transverse energy^31^. The plots are shown in Fig. 5(a,b) for a laser propagation distance of 3.5 mm and 5.5 mm, respectively. The comparison of Fig. 5(a,b) shows that the electrons have strong coupling with the transverse laser field. A considerable portion of the electrons obtains energy from the transverse laser electric field, which is evidence of resonantly excited betatron x-ray emission. Direct laser acceleration and related resonant betatron movement in the laser plasma wakefield have been investigated in theoretical studies^31^,^32^, which provided insights into the detailed dynamics of laser electron coupling. However, because the topic of the present work is the benefit of ionization injection for betatron x-ray enhancement, we focus on a discussion of how the ionization-injection process is better than self-injection with respect to electron early injection.

Ionization injection is a type of fast injection, which enables the electron betatron oscillations to rapidly change from non-resonant to resonant. For an electron born near the peak of the laser field in the plasma wake, the trapping condition can be written as 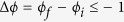
21,33, where 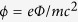
is the normalized electrostatic potential of the plasma wave, and the subscripts *i* and *f* denote the electron initial ionization and final injection point in the wake, respectively. In the case of a well matched bubble, the maximum wake potential 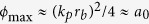, where 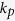 is the plasma wave number and 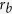 is the bubble radius. Thus, if the electron is born at the point where the wake potential is at the maximum, it can be injected when 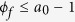. In our case, 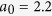, the electron has a high probability of being injected with 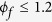. This suggests a fast injection process because the electron can be injected at the middle of bubble and does not need to slip to the bubble tail, where the wakefield potential approaches zero. This result is confirmed by our simulations. As shown in Fig. 5(c,d), the wakefield potentials of the ionization and injection point are 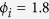 and 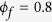, which satisfy the injection conditions. The electron is born at 113 μm and injected at 314 μm, corresponding to a fast injection process of 0.66 ps. In case of self-injection, the electron injection is strongly influenced by the nonlinear laser evolving process in plasma^34^,^35^,^36^. The electron injection is usually found at the tail of the bubble, where the wakefield potential is minimum. Thus, it takes a long time for the electron to be accelerated to catch up with the laser pulse, which may have already drastically decayed because of pump depletion and pulse diffraction. For example, in ref. 13, a 3-cm-long capillary was used to achieve the betatron resonant condition and this condition was easily mismatched when the plasma density was slightly changed. By contrast, in the ionization injection scheme, electrons are injected quickly and close to the laser pulse, so that they can catch up with the laser pulse at an earlier time and perform betatron resonant oscillation well before the laser intensity decreases. Therefore, the fast ionization-injection process is beneficial for more effectively stimulating resonant betatron oscillations. This faster injection speed and better injection position for electrons to catch up with the laser pulse were confirmed by comparison of the simulation results for nitrogen and helium shown in Fig. 4. The higher x-ray photon energy and photon yield with nitrogen gas compared with helium gas in our experiment and previous works^12^,^14^ support this conclusion. Owing to the fast injection process in nitrogen, electron dephasing occurs earlier and results in a lower maximum electron energy compared with helium, which is in agreement with the experimental results in Fig. 2(i,j).

In conclusion, we demonstrate the generation of intense betatron hard x-ray radiation via ionization injection for the first time using 100 TW laser pulses incident into a pure nitrogen gas jet. The betatron x-ray has a single pulse photon yield of 8 × 10^8^ and has 10^8^ photons greater than 110 keV. The photon energy (critical energy of 75 keV) is much higher and the photon flux is 10 times larger than when using helium gas (i.e., without ionization injection) under the same laser conditions. Considering the x-ray duration of ~10 fs, the estimated peak brilliance of the x-ray source is ~10^23 ^photons/(mm^2^ mrad^2^ 0.1%bandwidth), which is comparable to the 3^rd^ generation synchrotron light source but is in an unprecedented energetic x-ray region. The ionization-injection scheme stimulated by nitrogen gas shortens the time for the injected electrons to catch up with the laser pulse, which increases the efficiency of the resonant betatron oscillations. This experiment proved that ionization injection is a simple and convenient method to enhance the photon yield and energy of betatron x-ray beams from laser-wakefield acceleration. By better frequency matching^31^,^32^ and utilizing PW class laser pulses, stable gamma-ray beams with photon number greater than 10^10^ /pulse are expected from a single-stage nitrogen gas jet.

## Methods

### Laser system

The experiment was performed using a 100 TW laser system at the Key Laboratory for Laser Plasmas at Shanghai Jiao Tong University. In the experiment, the system delivered 40 fs pulses with energy up to 3 J. The pulses were focused by an f/20 off-axis-parabola (OAP) to a vacuum focal spot with a 1/e^2^ radius of w_0_ = 21 μm containing 50% energy. The resultant laser peak intensity reached 1.0 × 10^19^ W/cm^2^, corresponding to normalized vector potential a_0_ = 2.2, where *a*_0_ = 8.6 × 10^−10^λ[μm]I^1/2^[W/cm^2^].

### Gas jet

A 1.2 mm × 10 mm supersonic gas jet nozzle was used, which was capable of producing supersonic gas flow with a Mach number ~ 5. The gas jet can generate well-defined uniform gas density profiles in the range of 1 × 10^18 ^cm^−3^~ 3 × 10^19 ^cm^−3^ by changing the gas stagnation pressure^34^, for which the density information is based on the hydrodynamic calculations reported by Hosokai^37^.

### Diagnostics of electron beams and X-ray radiation

The electron beams emitted from the gas jet were dispersed by a 16 cm-long dipole magnet with magnetic field strength 0.9 T. A combination of 4 image plates (IPs) (Fuji Film SR series) covered with 13 μm Al foil was placed behind the magnet to record the electron and x-ray signals simultaneously. The IP was calibrated to measure the electron beam charge and betatron x-ray photon yield^38^,^39^. The electron energy was determined by different displacements on the consecutive IPs as in ref. 22. Because the IP (SR series) contains a 160 μm thick ion layer, it was also be used as x-ray filters as well as tungsten filters, as shown in Fig. 1. Thus, the photo-stimulated luminescence (PSL) values deposited on each IP correspond to x-ray photon energy greater than 7 keV, 36 keV, 52 keV and 110 keV, respectively. For comparison, nitrogen and helium gases were used in the same experiment. Because there is a large ionization potential gap between the L-shell electrons and K-shell electrons of the nitrogen atom, the charge state for the background nitrogen ions is assumed to be 5+.

### PIC simulation

The simulations were performed using the KLAPS code^40^,^41^, and the tunnel-ionization model^42^ was adopted for field ionization. We used a simulation box of 80 × 160 μm^2^ and adopted longitudinal and transverse resolutions of Δz = 0.025 μm and Δx = 0.2 μm, respectively. A p-polarized 800 nm laser pulse with a_0_ = 2.2 was focused to a radius of w_0_ = 21 μm at 100 μm after the front edge of the nitrogen gas. The pulse had a Gaussian transverse profile and sine-square longitudinal shape with pulse duration of τ_FWHM_ = 45 fs. The neutral nitrogen gas had a 100 μm up-ramp at the front and a 100 μm down-ramp at the rear side, and a 1-cm-long plateau. The neutral densities utilized in the simulation were 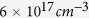 for nitrogen and 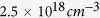 for helium, corresponding to background plasma densities of 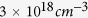 and 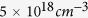, respectively.

## Additional Information

**How to cite this article**: Huang, K. *et al.* Resonantly Enhanced Betatron Hard X-rays from Ionization Injected Electrons in a Laser Plasma Accelerator. *Sci. Rep.* **6**, 27633; doi: 10.1038/srep27633 (2016).
